# Oxytocin Signaling Defines a Path toward Individualized Stroke Therapy

**DOI:** 10.1021/acscentsci.6c00633

**Published:** 2026-04-20

**Authors:** Long Lin, Yu-Hui Lin

**Affiliations:** Department of Clinic Pharmacology, School of Pharmacy, 12461 Nanjing Medical University, Nanjing 211166, China

## Abstract

Estrogen-driven oxytocin signaling confers superior neuroprotection in female mice, highlighting a sex-aware strategy for enhancing stroke recovery.

## Graphical abstract

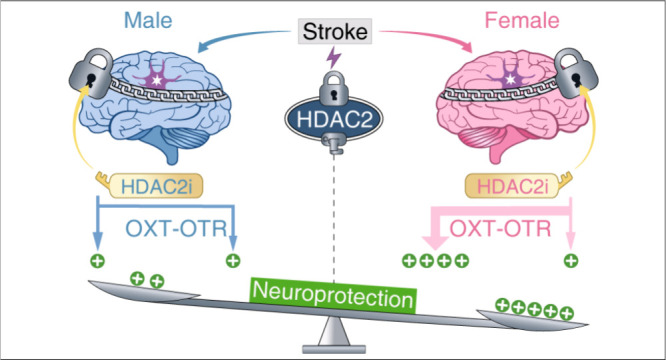

Despite extensive effort, the translation of neuroprotective strategies for ischemic stroke have been stymied, failing to bridge the gap between preclinical promise and tangible clinical benefits.^1,2^ A primary contributor to this impasse is the systemic oversight of biological sex, a pervasive yet underappreciated obstacle.^3^ Notwithstanding the mounting evidence of sex-specific molecular and cellular responses to stroke, neuroprotective drug development has largely persisted under the flawed premise of a sex-neutral landscape. This ‘one-size-fits-all’ paradigm risks bypassing sex-dimorphic mechanisms that may offer distinct therapeutic windows. In a recent issue of *ACS Central Science*, Fang, Geng, Yang, and colleagues challenged these conventions by identifying an epigenetically regulated, sex-dependent neuroprotective pathway orchestrated by oxytocin signaling. Their work establishes a transformative framework where biological sex is reconceptualized not as a source of confounding noise, but as a fundamental determinant of intrinsic resilience following stroke (Figure 1).^4^

**Figure 1:**
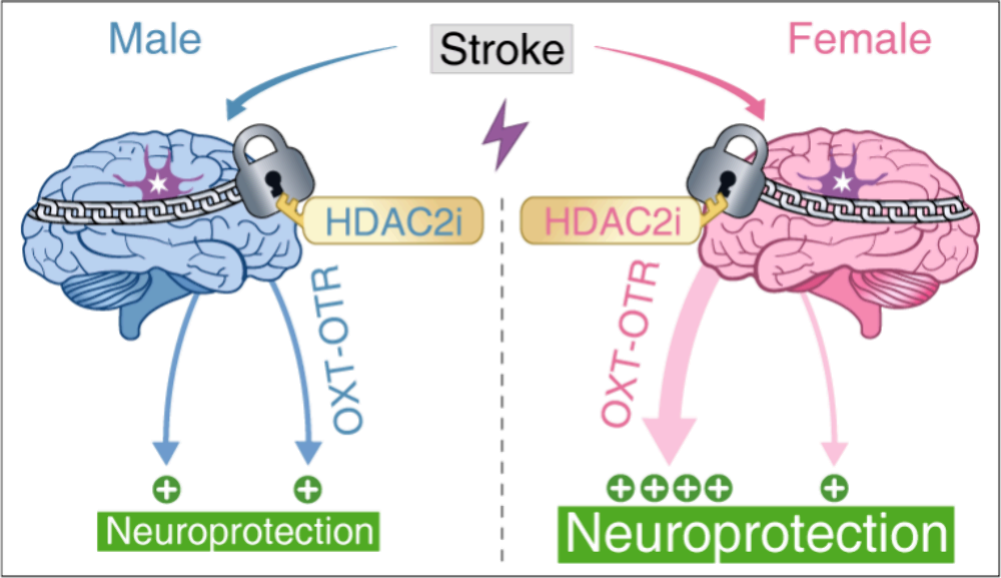
In both male and female brains, ischemic stroke triggers HDAC2-mediated functional repression, exerting a chain-mediated constraint around the site of injury. HDAC2 inhibition relieves this repression, conferring neuroprotection in both sexes. While this protective effect is modest in males, HDAC2 inhibition in females additionally engages and amplifies the OXT-OTR signaling axis, leading to superior neuroprotection.

To investigate sexual dimorphism in neuroprotection, the authors leveraged both *in vitro* oxygen-glucose deprivation/reoxygenation (OGD/R) and *in vivo* endothelin-1 (ET-1)-induced stroke models, coupled with the targeted disruption of HDAC2 via pharmacological and genetic approaches. Consistent with previous reports, HDAC2 modulation confers neuroprotection following stroke, significantly mitigating neuronal damage while enhancing cerebral blood flow and functional recovery.^5^ Notably, these neuroprotective effects are markedly more pronounced in females, highlighting a distinct sex-dependent response.^4^ Mechanistically, HDAC2 suppression triggers an upregulation of the oxytocin-oxytocin receptor (OXT-OTR) signaling axis, which coincides with the attenuation of neuroinflammation and oxidative stress.^4^ By linking epigenetic regulation to neurohormonal responses, the authors reposition the OXT-OTR pathway from a mere downstream correlate to a pivotal driver of sex-specific neuroprotection.^6^

Beyond simply preserving neural tissue, HDAC2 inhibition uncovers a dormant regenerative pathway centered on endogenous oxytocin signaling.

Beyond demonstrating the neuroprotective efficacy of HDAC2 inhibition, this study recasts HDAC2 as an epigenetic brake that restricts endogenous repair. Rather than operating through a single downstream effector, HDAC2 functions as a systemic brake on a broader neuroprotective program that is primed to be unlocked in a sex-dependent manner after injury. The alleviation of this epigenetic brake unleashes an endogenous recovery program, culminating in the robust upregulation of the OXT-OTR axis.^4^ Crucially, this response is sex-divergent, with females exhibiting a markedly more pronounced signaling amplification. The authors further trace this sex bias to estrogen-dependent modulation, revealing a mechanistic axis where epigenetic control, neurohormonal signaling, and gonadal hormones converge.^4^ Rather than serving as a passive permissive factor,^7^ estrogen acts as a dynamic potentiator that synergistically augments the OXT-OTR axis to drive superior neuroprotection upon HDAC2 suppression. Beyond simply preserving neural tissue, HDAC2 inhibition uncovers a dormant regenerative pathway centered on endogenous oxytocin signaling. Collectively, these findings shift the paradigm from ‘HDAC2 as a target’ to ‘HDAC2 as a gatekeeper’, whose inhibition derepresses a sex-biased, hormone-sensitive resilience program intrinsic to the injured brain.

By interrogating a central dogma in stroke research, this study upends the premise of sex-neutral neuroprotection, challenging the traditional pursuit of ‘one-size-fits-all’ therapeutics. Rather than a background variable to be adjusted away, biological sex serves here as a mechanistic key that unlocks a strategy for selectively amplifying neuroprotection. The findings suggest that therapeutic success hinges not on the discovery of a singular panacea, but on the strategic mobilization of innate recovery pathways tailored to a patient’s unique biological profile. From a chemical biology perspective, this study underscores the transformative potential of tuning epigenetic regulators to rewire endogenous neurohormonal signaling. By targeting HDAC2, the authors effectively translate chromatin-level modulation into systems-level physiological outcomes, bridging the scales between molecular epigenetics and circuit-level neuroprotection. This integration paves the way for a new generation of therapies that transcend merely halting injury, moving instead toward actively reprogramming the brain’s intrinsic resilience mechanisms.

Rather than a background variable to be adjusted away, biological sex serves here as a mechanistic key that unlocks a strategy for selectively amplifying neuroprotection.

Notwithstanding the study’s transformative potential, the path toward clinical translation warrants rigorous evaluation across several critical dimensions. First, while the ET-1-induced stroke model provides robust experimental stability and reproducibility, it may not fully encapsulate the pathophysiological heterogeneity inherent in diverse ischemic stroke subtypes.^8^ As the authors acknowledge, ET-1 exerts pleiotropic effects beyond localized vasoconstriction, including direct modulation of neuronal and glial activity that could confound both injury progression and recovery dynamics. To bolster the translational validity of these findings, it is essential to determine whether the OXT-OTR-mediated recovery pathway is conserved across diverse ischemic paradigms. Second, the estrogen-contingent nature of this mechanism introduces complexities for clinical implementation. Given that stroke predominantly affects postmenopausal women, a demographic characterized by profound estrogen depletion, the therapeutic reach of an estrogen-dependent axis requires further elucidation.^9^ This identifies a critical research gap, defining the mechanistic feasibility of engaging this OXT-OTR centered axis across diverse endocrine landscapes. To circumvent these barriers, the authors propose several strategic interventions, including transient estradiol priming, the deployment of selective OXT-OTR agonists or blood-brain barrier-permeant analogs, and the adoption of age- and hormone-stratified experimental frameworks.^10^ Finally, the discovery of the HDAC2-OXT-OTR axis serves as a compelling proof-of-concept, raising the question of whether other latent, sex-dimorphic recovery programs remain to be harnessed beyond the oxytocinergic system. Notably, it remains to be determined whether male-specific neuroprotective pathways exist that function independently of those identified in females. Beyond confirming the clinical relevance of this signaling pathway, the primary translational hurdle involves tailoring its application to account for the interplay between age, hormonal status, and the specific stroke context. Uncovering these complementary mechanisms will be pivotal for advancing a framework of individualized neuroprotection, wherein multifaceted, context-dependent pathways can be selectively activated to optimize therapeutic outcomes.

Beyond confirming the clinical relevance of this signaling pathway, the primary translational hurdle involves tailoring its application to account for the interplay between age, hormonal status, and the specific stroke context.

In summary, the significance of this study transcends the mere observation of superior neuroprotective responses in females. Instead, it reconceptualizes a foundational principle: biological sex is not a confounding variable to be controlled, but a reservoir of actionable biology to be harnessed. By elucidating the epigenetic unlocking of sex-specific recovery programs, Fang, Geng, Yang, and colleagues pioneer a paradigm for stroke therapy: one that embraces, rather than obscures, the fundamental heterogeneity of the ischemic brain.
